# Measuring the health literacy of the Upper Midwest

**DOI:** 10.5195/jmla.2017.105

**Published:** 2017-01

**Authors:** Caitlin J. Bakker, Jonathan B. Koffel, Nicole R. Theis-Mahon

## Abstract

**Objectives:**

Health literacy—the ability to obtain, process, and understand basic health information—is a major determinant of an individual’s overall health and health care utilization. In this project, the authors examined predictors of health literacy levels, including numeracy and graphic literacy, among an adult population in the Upper Midwest.

**Methods:**

The research was conducted at the Minnesota State Fair. Three previously validated scales were used to assess health literacy: Newest Vital Sign, the General Health Numeracy Test, and questions from Galesic and Garcia-Retamero’s Graph Literacy Scale. Demographic information—such as age, educational attainment, zip code, and other potential predictors and modifiers—was collected. Multivariate linear regression was conducted to examine the independent effects of educational attainment, race, ethnicity, gender, and rural or urban location on overall health literacy and scores on each of the individual instruments.

**Results:**

A total of 353 Upper Midwest residents completed the survey, with the majority being white, college-educated, and from an urban area. Having a graduate or professional degree or being under the age of 21 were associated with increased health literacy scores, while having a high school diploma or some high school education, being Asian American, or being American Indian/Alaska Native were associated with lower health literacy scores.

**Conclusion:**

Advanced health literacy skills, including the ability to calculate and compare information, were problematic even in well-educated populations. Understanding numerical and graphical information was found to be particularly difficult, and more research is needed to understand these deficits and how best to address them.

## INTRODUCTION

Health literacy is the “the degree to which individuals can obtain, process, and understand the basic health information and services they need to make appropriate health decisions” and effectively function in the health care environment [1]. These skills are central to patient-centered care as they inform the ability to engage in the decision-making process, such as deciding when an injury can be treated at home versus when an injury requires a trip to urgent care or understanding how and when an antibiotic should be taken.

Between one-third and one-half of adult Americans are estimated to have low health literacy [1, 2], while recent research from the National Center for Education Statistics notes that only 12% of American adults show the highest level of proficiency on a literacy scale, and only 9% of adults show the highest level of proficiency when considering numeracy (the ability to make basic calculations and understand relationships) [3]. Lower rates of health literacy have been found among the elderly, minority populations, persons of limited financial means, and those with less than a high school education [2]. Low health literacy is associated with limited understanding of medical information, poor health outcomes, and poor use of health care services, including increased hospitalization and emergency care [4, 5], decreased use of preventative services [6], poor health behaviors [7, 8], and inability to take medication as prescribed [9, 10].

In addition to the ability to read and interpret textual information, definitions of health literacy have expanded to incorporate numeracy and graph literacy (the ability to interpret graphs, charts, and similar graphics). Poor numeracy is associated with inaccurate estimation of risk when considering treatment options and an unwillingness to adhere to medication regimens [11], broadening the potential negative impact of low health literacy. As Furci and O’Donnell note, “our patients are complex beings composed not only of body, but also of mind and spirit. It is this complexity that can make patients a challenge to treat” [12].

Graphs and charts have been proposed as one way to make risk information and other numerical information easier for patients to understand. The underlying idea is that these graphs “facilitat[e] the nonnumeric, holistic, and gist-based translation from quantitative to qualitative meaning” [13]. The effectiveness of graphical representations depends on the patient’s ability to accurately interpret the information. Previous research with emergency department patients shows that health literacy and numeracy are poorly correlated [9], but it is unclear whether this holds true for the general population and their specific relationships with graph literacy.

Despite the requirement to include communication skills training in both graduate and undergraduate medical education [14, 15], evidence suggests that clinical faculty may be unprepared to teach and evaluate such skills [16, 17]. A growing body of literature notes that health literacy and effective communication skills are inconsistently or inadequately addressed in medical education [18–22]. Studies have further documented residents’ and clinicians’ use of ineffective communication strategies [23–26].

With their knowledge of resident and physician instruction methods, information appraisal, and patient education resources, librarians are well positioned to train clinicians in understanding the challenges of health literacy and effectively conveying information. The aim of this study was to examine predictors of and potential correlations between different aspects of health literacy among people living in the Upper Midwest.

## METHODS

### Design and participants

The authors conducted a health literacy survey at the 2015 Minnesota State Fair over 3 days in August and September among adults with a working knowledge of the English language. The Minnesota State Fair is the largest state fair in the United States, with over 1.7 million attendees in 2015 [27]. This setting was chosen because it allowed us to rapidly recruit and reach a population who might not otherwise participate in university- or clinic-based research. Participants received a University of Minnesota backpack, valued at approximately $2, as an incentive. The survey took approximately 15 minutes to complete and was offered in paper or on a tablet. This research was approved as exempt by the Institutional Review Board (IRB) at the University of Minnesota.

After reviewing the information and consent sheets, participants were asked to complete the survey and to provide demographic information. We had filed a waiver of documentation of informed consent with the IRB prior to the research, meaning that signed consent forms were not necessary in this research. Our survey was developed from 3 separate instruments to assess 3 different aspects of health literacy. The instrument, which blended components of the 3 previously validated surveys, was found to be internally consistent (Cronbach’s alpha coefficient=0.84).

Health literacy was assessed using the Newest Vital Sign (NVS), a commonly used instrument for assessing health literacy [28]. NVS includes 6 questions related to interpreting nutritional labels and takes approximately 3 minutes to complete. Its internal consistency was established in a previous study with a Cronbach’s alpha coefficient of 0.76 [28].

The General Health Numeracy Test (GHNT-6) was used to assess numeracy [29]. This is a 6-item test that takes approximately 5 minutes to complete. The instrument consists of word problems and 1 question related to the interpretation of nutritional labels. The internal consistency of GHNT-6 was previously established with a Kuder-Richardson coefficient of 0.77 [29].

Eight questions from the Graph Literacy Scale (GLS) developed by Galesic and Garcia-Retamero were selected to assess graphic literacy [30]. The GLS was developed to assess an individual’s ability to read, compare, and interpret data shown in a chart. The questions were estimated to take approximately 5 minutes to complete, and the complete scale, which has 13 questions, was found to be internally consistent in previous research with a Cronbach’s alpha of 0.85 [30].

### Data analysis

In addition to the responses to the survey, demographic data on gender, educational attainment, ethnicity, race, and age range were recorded. Five-digit zip codes were recorded and used in conjunction with 2013 urban influence codes (UIC) codes to determine rural and urban locations [31]. Participants who provided zip codes outside of the Upper Midwest region were excluded from our analysis. We defined the Upper Midwest region as North Dakota, Minnesota, Wisconsin, and Iowa. We did not receive responses from individuals from other states in the Upper Midwest, such as South Dakota or Nebraska.

Surveys completed on paper were entered into an electronic system. Double data entry was conducted on 52 of the 256 paper surveys to identify likelihood of discrepancy. No discrepancies were found. Overall health literacy scores were calculated as the percent of the 20 total questions answered correctly.

We conducted multivariate linear regressions to examine the independent effects of educational attainment, race, ethnicity, gender, and rural or urban location on overall health literacy and scores on each of the individual instruments. We dummy-coded nonbinary categorical variables (i.e., all but rural versus urban location) as binary variables (e.g., 31–40 years old versus a different age group), which allowed us to compare each group against a reference group. We designated the reference group as women, non-Hispanic, white, 41–50 years old, with a bachelor’s degree, and living in an urban area, as these were the most common respondents in each category. When conducting the analyses, we employed list-wise deletion, only analyzing responses from participants who answered all background and demographics questions.

## RESULTS

Over 3 days, 373 surveys were completed, and 353 ultimately analyzed. Excluded surveys were those completed by individuals located outside of the Upper Midwest region. Respondents were primarily white (n=290, 82%), not Hispanic or Latino (n=325, 92%), and from an urban area (n=275, 78%). The majority of respondents (n=216, 61%) had obtained a bachelor’s degree or a graduate or professional degree. Overall participant characteristics and average scores are presented in Table 1. Statistical significance in comparison to the reference group is also noted. Three hundred ten participants responded to all demographics questions and could be included in the regression analyses (Table 2). The fit for each model was generally low (*r*^2^ ranging from 0.17–0.23) but significant (*p*<0.001 in all models).

**Table 1:** Participant demographics and instrument scores

				% Correct		
	n	%	Overall	NVS	GHNT-6	GLS
Gender						
Female	224	63.5%	72.4%	87.5%	59.9%	70.0%
Male	117	33.1%	73.6%	77.5%*	67.8%*	74.9%*
Unknown/other	12	3.4%	54.6%	75.0%	48.6%	43.8%
Education						
Graduate or professional degree	80	22.7%	80.4%*	89.2%*	72.5%*	79.8%*
Bachelor’s degree	136	38.5%	73.5%	84.3%	63.5%	72.8%
Associate’s degree	32	9.1%	70.0%	82.8%	57.3%	66.8%
Some college	57	16.1%	69.9%	83.3%	59.7%	67.5%*
High school diploma or GED	32	9.1%	65.0%*	79.2%	52.1%*	64.1%
Some high school	5	1.4%	35.0%*	46.7%*	20.0%*	37.5%*
Unknown	11	3.1%	51.8%	72.7%	45.5%	40.9%
Age						
18–20 years	27	7.6%	73.2%*	86.4%	63.0%*	70.8%*
21–30 years	71	20.1%	75.4%	83.6%	69.5%*	73.6%
31–40 years	37	10.5%	76.1%	84.2%	67.6%	76.4%
41–50 years	80	22.7%	71.4%	85.0%	58.6%	70.8%
51–60 years	79	22.4%	71.0%	83.5%	59.5%	70.3%
61–70 years	39	11.0%	73.3%	83.8%	61.5%	71.8%
71 and over	8	2.3%	56.9%	72.9%	45.8%	53.1%*
Unknown	12	3.4%	57.9%	77.8%	52.8%	46.9%
Race						
White	290	82.2%	74.4%	86.0%	63.8%	73.3%
Black or African American	5	1.4%	51.0%	60.0%	40.0%	52.5%
Asian	27	7.6%	68.2%*	73.5%*	63.6%	67.6%*
American Indian/Alaska Native	5	1.4%	40.0%*	50.0%*	36.7%	35.0%*
Multiracial	7	2.0%	75.0%	88.1%	64.3%	73.2%
Other	7	2.0%	58.6%	76.2%	45.2%	55.4%
Unknown	12	3.4%	55.8%	79.2%	47.2%	44.8%
Ethnicity						
Hispanic or Latino	9	2.5%	66.7%	83.3%	55.6%	62.5%
Not Hispanic or Latino	325	92.1%	73.0%	84.5%	62.8%	71.9%
Unknown	19	5.4%	60.0%	71.9%	54.4%	55.3%
Location						
Urban	275	77.9%	73.3%	84.2%	63.0%	72.4%
Non-urban	46	13.0%	75.3%	88.0%	65.2%	73.4%
Unknown	32	9.1%	58.1%	73.4%	50.0%	52.7%
Total	353	100.0%	72.2%	83.8%	62.1%	70.7%

* Statistically significant at *p*<0.05 in comparison to a reference group (women, non-Hispanic, white, 41–50 years old, with a bachelor’s degree, and living in an urban area).

**Table 2:** Summary of multivariate linear regression analysis

		Overall			NVS			GHNT-6			GLS	
	*B*	*SE B*	*β*	*B*	*SE B*	*β*	*B*	*SE B*	*β*	*B*	*SE B*	*β*
Constant	72.931	2.514	—	89.363	2.998	—	58.351	3.755	—	71.596	2.642	—
Gender												
Female	Reference											
Male	1.593	2.168	0.039	−9.568	2.585	−0.201	8.353	3.237	0.143	5.297	2.277	0.125
Education												
Bachelor’s degree	Reference											
Graduate or professional degree	9.096	2.587	0.206	8.134	3.085	0.157	9.921	3.863	0.156	9.365	2.718	0.202
Associate’s degree	−0.077	3.779	−0.001	−0.088	4.507	−0.001	−3.045	5.644	−0.031	−1.661	3.970	−0.023
Some college	−5.069	3.113	−0.100	0.507	3.712	0.009	−8.343	4.649	−0.114	−6.941	3.270	−0.131
High school diploma or GED	−9.390	3.968	−0.146	−4.001	4.731	−0.053	−16.773	5.925	−0.181	−7.699	4.168	−0.114
Some high school	−37.550	9.272	−0.222	−33.242	11.057	−0.167	−51.808	13.848	−0.213	−30.208	9.741	−0.170
Age												
41–50 years old	Reference											
18–20 years old	10.616	4.560	0.154	4.107	5.437	0.051	17.365	6.810	0.175	10.309	4.790	0.143
21–30 years old	5.008	3.004	0.108	0.416	3.582	0.008	11.026	4.487	0.165	4.127	3.156	0.085
31–40 years old	3.141	3.683	0.051	−0.959	4.392	−0.013	5.949	5.500	0.067	4.179	3.869	0.064
51–60 years old	−1.727	2.909	−0.038	−3.255	3.469	−0.061	0.369	4.345	0.006	−2.098	3.057	−0.044
61–70 years old	1.153	3.650	0.019	−2.110	4.353	−0.029	3.828	5.451	0.044	−1.447	3.835	−0.023
71+ years old	−12.256	6.888	−0.095	−10.367	8.214	−0.69	−9.593	10.287	−0.052	−16.075	7.237	−0.119
Race												
White	Reference											
Black or African American	−3.852	12.399	−0.016	−4.256	14.785	−0.015	−2.737	18.517	−0.008	−3.204	13.026	−0.013
Asian	−11.867	3.771	−0.172	−14.203	4.497	−0.176	−8.822	5.632	−0.089	−12.676	3.962	−0.176
American Indian/Alaska Native	−44.403	10.456	−0.228	−63.358	12.468	−0.277	−24.056	15.615	−0.086	−44.377	10.985	−0.217
Multiracial	3.642	7.798	0.028	5.575	9.299	0.037	3.271	11.645	0.018	2.246	8.192	0.017
Other race	−6.543	7.489	−0.047	4.471	8.930	0.027	−6.757	11.184	−0.034	−14.918	7.867	−0.103
Ethnicity												
Not Hispanic or Latino	Reference											
Hispanic or Latino	−2.726	7.186	−0.023	−5.350	8.569	−0.038	0.256	10.731	0.981	−2.961	7.549	−0.023
Location												
Urban	Reference											
Rural	1.642	2.852	0.031	1.947	3.401	0.568	2.790	4.259	0.036	1.113	2.996	0.020
*r^2^*		0.232			0.209			0.172			0.229	
*F*		4.388			3.841			3.020			4.310	

Note: Constant is a reference group (women, non-Hispanic, white, 41–50 years old, with a bachelor’s degree, and living in an urban area).

In comparisons with the reference group of 41–50 year old white, non-Hispanic, urban-dwelling females with a bachelor’s degree, education, race, and age were found to have statistically significant differences when considering overall score. Ethnicity and location were not statistically significant predictors of overall score or scores on any of the individual instruments that constituted the survey. Although not a significant predictor of overall score (β=0.039, *p*=0.463), being male significantly predicted scores on the NVS (β=−0.201, *p*<0.001), GHNT-6 (β=0.143, *p*=0.010), and GLS (β=0.125, *p*=0.021). Being male decreased NVS score by 9.57 percentage points but increased GHNT-6 and GLS scores by 8.35 and 5.30 points, respectively. Age was also a significant predictor, with being 18–20 years old increasing overall score by 10.62 points (β=0.206, *p*=0.021), increasing GHNT-6 score by 17.37 points (β=0.175, *p*=0.011), and increasing GLS score by 10.31 points (β=0.143, *p*=0.032). Being over the age of 70 significantly decreased GLS score by 16.08 points (β=−0.119, *p*=0.027). Being 21–30 years old significantly increased GHNT-6 score by 11.03 points (β=0.165, *p*=0.015). Age did not significantly predict NVS score.

Being Asian American (β=−0.172, *p*=0.002) or American Indian/Alaska Native (β=−0.228, *p*<0.001) decreased overall score by 11.87 and 44.40 points, respectively. Being Asian American significantly decreased NVS (β=−0.176, *p*=0.002) and GLS (β=−0.176, *p*=0.002) scores by 14.20 and 12.68 points, respectively, although it did not significantly predict GHNT-6 score (β=−1.567, *p*=0.118). Similarly, being American Indian/Alaska Native significantly decreased NVS (β=0.277, *p*<0.001) and GLS (β=−0.217, *p*<0.001) scores by 63.36 and 44.38 points, respectively, but it did not significantly predict GHNT-6 score (β=−1.541, *p*=0.125). Being a member of other racial groups did not significantly predict overall score or scores on individual instruments.

Having a graduate or professional degree (β=0.206, *p*=0.001), a high school diploma (β=−1.46, *p*=0.019), or less than a high school diploma (β=−0.222, *p*<0.001) were significant predictors of overall score. Having a graduate or professional degree increased overall score by 9.10 points, while having a high school diploma decreased overall score by 9.39 points, and having less than a high school diploma decreased overall score by 37.56 points. Having an associate’s degree (β=−0.001, *p*=0.984) or some college education (β=−0.100, *p*=0.105) was not a significant predictor of overall score. In the analysis of individual instruments in the survey, the upper and lower ends of educational attainment remained significant predictors of GLS, NVS, and GHNT-6 scores. Having a graduate degree significantly predicted NVS (β=0.157, *p*=0.009, 8.13 point increase), GHNT-6 (β=0.156, *p*=0.011, 9.92 point increase), and GLS (β=0.202, *p*=0.001, 9.37 point increase) scores. Having less than a high school education also significantly predicted NVS (β=−0.167, *p*=0.003, 33.24 point decrease), GHNT-6 (β=−0.213, *p*<0.001, 51.81 point decrease), and GLS (β=−0.170, *p*=0.002, 30.21 point decrease) scores. Having completed some college education significantly predicted GLS score (β=−0.131, *p*=0.035, 6.94 point decrease), while having attained a high school diploma or GED significantly predicted GHNT-6 score (β=−0.181, *p*=0.005, 16.77 point decrease). Significant correlations existed between all instruments. GHNT-6 and GLS scores were strongly correlated with overall scores (*rs*=0.875 and 0.833) and strongly correlated with each other (*r*=0.617). NVS scores were strongly correlated with overall scores (*r*=0.654) but only weakly or moderately correlated with GHNT-6 or GLS scores (*rs*=0.453 and 0.382).

## DISCUSSION

We found that three factors were significant predictors of overall health literacy scores: educational attainment, age, and race. Specifically, having completed a graduate or professional degree or being under the age of twenty-one was associated with higher health literacy, while having a high school education or less than a high school diploma, being Asian American, or being American Indian/Alaska Native was associated with lower health literacy. However, only having completed a graduate or professional degree or having completed less than a high school diploma significantly predicted both the overall score and all individual instrument scores.

Elderly populations have previously been found to have lower levels of health literacy [2]. However, we found that being over the age of seventy was only associated with lower scores in graphic literacy. Being under the age of thirty was a significant predictor of higher numeracy scores, while being under the age of twenty-one was a significant predictor of higher graphic literacy. That younger adults show higher scores on numeracy scales has been previously established, although previous studies have reported that the elderly “demonstrate significantly lower numeracy” [32], which was not supported by our results. The assessment of health literacy levels among older adults has proved somewhat controversial, as notably different levels of adequacy and inadequacy are found when using different measures [33].

Being Asian American or American Indian/Alaska Native was a significant predictor of lower overall health literacy score as well as all scores on all individual instruments except the numeracy scale. Although our small sample sizes limit the conclusions that can be drawn, these significant associations should not be dismissed entirely, as these 2 groups are particularly important in the Upper Midwest. Between 2010 and 2015, Asian Americans were the fastest growing racial minority in these 4 states, increasing from 410,884 to 511,815 [34]. While the American Indian and Alaska Native population has not grown as dramatically, it remains substantial in these regions, with populations of indigenous people being higher in Minnesota and North Dakota than the national average [34]. That only 2 of the minority groups were significant predictors of low health literacy is somewhat surprising. In a systematic review of 85 studies, it was found that “[t]he rate of black subjects was significantly associated with the rate of low literacy” [35], although it was noted that several confounding factors, such as educational attainment, might have influenced those results.

Broad categorizations are limited in that they do not consider factors unique to distinct ethnic groups and, particularly in the case of Asian Americans, may be overly simplistic. As Kim and Keefe note, there are between twenty-seven and thirty-two Asian American groups in the United States, each of which may have its own economic, cultural, linguistic, social, and political context impacting health [36]. This project did not aim to provide the granularity necessary to examine predictors of health literacy and behaviors among the diverse ethnic groups within these broader categories, as there was already a relatively extensive body of literature available on these topics [36–39]. However, that membership in these substantial minority groups was found to be a significant predictor of poor health literacy should be considered in developing and implementing context-specific educational interventions. This finding may indicate the importance of considering cultural competence and its relationship to health literacy training. Further research is needed, however, to validate and examine the potential effect of race on health literacy.

While our findings that low health literacy was associated with lower levels of education supported the connection between academic achievement and health behaviors [35, 40], higher levels of educational attainment did not necessarily result in consistently high scores across all aspects of health literacy. At every level of educational attainment, the same pattern was noted: scores were highest on the NVS and lowest on the GHNT-6. In the case of individuals with graduate or professional degrees, the average NVS score was 89.2, but the average GHNT-6 score was only 72.5. Consistent with previous research, this result suggests that completing basic numeracy questions poses a challenge and that numeracy skills cannot be assumed by level of education [41]. Although the mean numeracy score among graduate degree holders was 10 points higher than the overall average of 62.1, 30% (n=24) of respondents with graduate degrees scored 50% or less on this scale. When examining responses to individual questions in the survey, questions that required comprehension of information, such as reading a number off a graph, appeared to be easier for respondents than questions that required calculations or inference. Given that participants had access to calculators, pens, and paper and were given unlimited time, one can also imagine the potential challenge of receiving and understanding such information verbally and in a stressful situation, such as when being seen in a doctor’s office or when making difficult decisions regarding treatment options.

Our findings echo Mayer’s Multimedia Learning Principle that “people learn better from words and pictures than from words alone” [42]. Strategies that incorporate multiple methods of communicating information have been shown to positively impact patient outcomes. One study showed that a pictogram-based intervention augmented with demonstration and teach-back methods reduced caregiver dosing errors [43]. A disease-management intervention that incorporates plain language, teach-back, and visual aids is associated with a greater likelihood of achieving prescribed health outcomes [44]. Previous research had indicated that health literacy interventions for medical students—including teaching plain language skills, teach-back techniques, and the use of universal precautions—lead to significant improvement in self-reported knowledge and planned behaviors [45]. Medical educators can assist residents and clinicians with using these strategies and developing these skills to effectively communicate health information to patients and caregivers. Brown and Bylund’s “Breaking Bad News” module, in their communication skills training workshop, outlined strategies for providing understandable information to patients, including avoiding jargon, using a variety of methods for conveying information, and offering written information [46]. Medical educators have also addressed these skills in a variety of formats, including role-playing [47], interactive workshops [48], and problem-based learning [49].

One approach to increase health literacy is to utilize and involve librarians in educating residents and physicians, particularly through community and faculty partnerships [50, 51]. For example, at the University of Minnesota, library integration into a required third-year course for all medical students has provided an opportunity to discuss the importance of plain language and health literacy. This session incorporates a graded component in which the students must translate findings from scholarly articles into patient education resources. Librarians at the University of Manitoba offer similar instruction to first-year family medicine residents during a half-day session that incorporates active learning elements, including administering health literacy screening tools such as NVS for clinical use [52]. Librarians at the University of Utah have been actively engaged in their university-wide Health Literacy Interest Group since 2006 and have hosted workshops and lectures on this topic. Recently, the libraries at University of Utah were involved in Healthi4U, a competition that encouraged students to create videos focused on health care topics in a variety of languages. The winning videos are now being broadcast on the patient education channel at University of Utah Health Care and through the Utah Educational Network’s television station [53]. However, despite these successful partnerships, literature on librarian-led educational interventions remains limited.

Consideration of all aspects of health literacy is imperative for clinicians as they engage patients in the decision-making process. More advanced skills, including the abilities to calculate and to compare information, remain problematic even for well-educated populations. Although there are ongoing attempts by medical educators to develop these skills in medical students, residents, and clinicians, increased librarian involvement could provide a beneficial perspective.

### Study limitations

This study employed non-probability sampling, and therefore, its sample is not representative of all individuals in the Upper Midwest. This is most notable when considering educational attainment. While 32.5% of American adults have a bachelor’s degree or higher [34], 61% of our study respondents held these same credentials. While this limits the generalizability of our findings, this overrepresentation is not unexpected in a self-selecting group, as individuals with higher educational attainment are more likely to participate in surveys [54]. Also, standard values for adequate health literacy have not been established for the GHNT-6 or GLS, which instead rely on percent correct or median split. Further research that includes systematic or stratified samples and standard values for numeracy and graphic literacy is necessary to establish a baseline of health literacy in the general population.
